# Optically induced mode splitting in self-assembled, high quality-factor conjugated polymer microcavities

**DOI:** 10.1038/srep19635

**Published:** 2016-01-19

**Authors:** Daniel Braam, Soh Kushida, Robert Niemöller, Günther M. Prinz, Hitoshi Saito, Takaki Kanbara, Junpei Kuwabara, Yohei Yamamoto, Axel Lorke

**Affiliations:** 1 Fakultät für Physik and CENIDE, Universität Duisburg-Essen, Lotharstraße 1, 47057 Duisburg, Germany; 2 Division of Materials Science and Tsukuba Research Center for Interdisciplinary Materials Science (TIMS), Faculty of Pure and Applied Sciences, University of Tsukuba, 1-1-1 Tennodai, Tsukuba, Ibaraki 305-8573, Japan

## Abstract

We investigate the whispering gallery modes (WGMs) of self-assembled single microspheres. They consist of a recently developed highly fluorescent *π*-conjugated copolymer and exhibit excellent optical properties with Q-factors up to 10^4^. Under continuous laser irradiation, we observe a splitting of the highly degenerate spherical WGMs into a multiplet of lines. Comparison with the calculated spectral response of a weakly distorted sphere shows that the optical excitation induces a change of the optical path length in the microcavity so that it resembles a prolate spheroid. The separation of the lines is given by the ellipticity and the azimuthal mode number. Measurements in various gaseous environments suggest that the distortion is caused by light induced oxidation of the polymer. Our findings show that photooxidation can be a beneficial mechanism for *in-situ* tuning of optically active polymer structures.

Optical microcavities play an important role in both basic and applied research due to their ability to confine light at extremely narrow resonance frequencies. This enables diverse applications, such as laser resonators^1^, photon pair generation^2^, and protein^3^ or on-chip single nanoparticle sensors^4^. Most of these microcavities consist of two constituents: A dielectric, which serves as a resonator, and a fluorescent dye, which ensures the optical activity. In contrast, *π*-conjugated polymers can combine the features of being a dye and a dielectric, so that only a single material is necessary. Furthermore, they easily form microcavities by self-assembly^5^,^6^. Finally, because of their good electrical properties, they may bridge the gap between conventional, passive optical resonators and organic light emitting devices. Thus, optically active structures made from *π*-conjugated polymers are very promising candidates for a variety of future photonic and optoelectronic devices.

Here, we report on sharp whispering gallery modes from single, high quality polymer microspheres, which exhibit quality (Q−) factors more than an order of magnitude higher than in previous studies^5^. After prolonged optical pumping, we observe a distinct splitting of the whispering gallery modes. Calculations within the framework of first order perturbation theory explain this splitting as an optically induced shape change of the originally spherical microresonator into a prolate spheroid. This lifts the azimuthal degeneracy and leads to a multiplet, where each line corresponds to a specific azimuthal mode number. Further investigation of this phenomenon, subjecting the microspheres to different gaseous environments, suggests that the shape change is caused by light induced oxidation of the polymer.

## Results and Discussion

**Figure 1:**
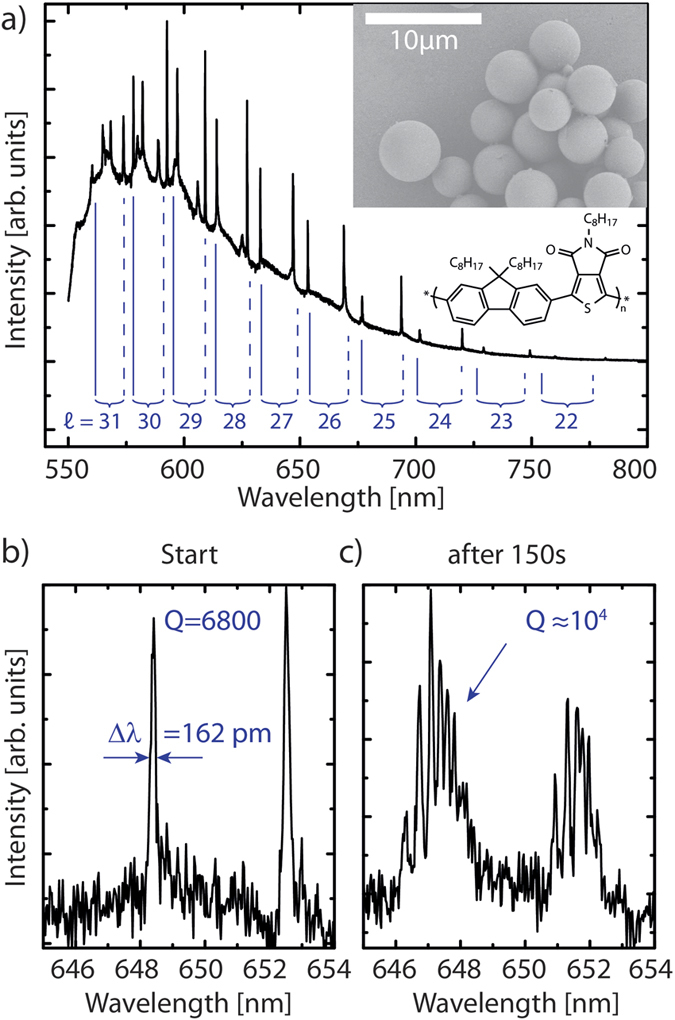
(**a**) Measured PL spectrum (top, black) of a single self-assembled sphere (radius 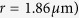, made from the *π*-conjugated alternating copolymer shown in the lower inset. Sharp whispering gallery modes are observed. Vertical lines are calculated positions of TE (dashed) and TM (solid) modes with angular momentum numbers 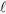. Upper inset: SEM image of typical spheres. (**b**) High resolution spectrum of the 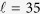 TE and 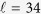 TM modes of a different sphere (radius 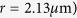. (**c**) Splitting of the modes, shown in (**b**), after 150 s optical excitation with 7.5 *μ*W.

Figure 1(a) shows the photoluminescence (PL) spectrum of a single microsphere of 1.86 *μ*m radius in the range between 550 and 800 nm. A broad emission is observed with a maximum around 575 nm. Superimposed are a set of sharp resonances, which are roughly equidistant in the inverse wavelength. As in previous studies^5^,^7^,^8^, these resonances can be attributed to whispering gallery modes within the microsphere with different angular momentum numbers 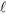. These modes can be calculated for a spherical resonator of a few micrometers^8^, taking the sphere diameter from optical microscopy and the dielectric function from ellipsometry 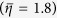. The blue vertical lines in Fig. 1(a) show the calculated positions for transverse electric (TE, dashed) and transverse magnetic (TM, solid) modes with 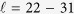 and radial mode number 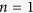. Here, we have slightly adjusted the radius of the sphere for the best fit. The calculated wavelength positions match nicely the experimental TE and TM modes, especially for the intermediate wavelength region. Small deviations are attributed to the wavelength-dependent dielectric function, which was averaged in the simulations for simplicity. Additional modes are observed, which can be attributed to higher order radial modes^9^ (*n* > 1).

In Fig. 1(b), two modes (TE, 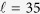, and TM, 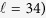 of another microsphere with 2.13 *μ*m radius are shown in high resolution. The full width at half maximum of these resonances is about 160 pm, which is a 10-fold improvement over microspheres made previously from different *π*-conjugated polymers^5^. Continuous illumination at 7.5 *μ*W (about 750 W/cm^2^) under ambient conditions leads to a shift and a splitting of all modes into a multiplet of sharp lines (see Fig. 1(c)). These exhibit linewidths, which are even narrower than those of the original peak and in fact limited by the optical resolution of the spectrometer. We estimate the original linewidth from a deconvolution of the experimental data and find a Q-factor of about 10^4^, which is the highest value ever reported for luminescent organic microspheres of this size. High Q-factors have been observed in dye doped polymer microspheres^9^, however, on spheres which were much larger than the present ones. Since surface roughness will set an upper limit for the Q-factor^10^, the observation of such narrow lines also shows the high morphological quality of our self-assembled polymer spheres. SEM images confirm the high surface quality (see inset in Fig. 1(a)). The fact that the linewidth decreases as the lines split up indicates that the original peak was a superposition of many almost degenerate lines and its width was inhomogeneously broadened by their incomplete overlap.

**Figure 2:**
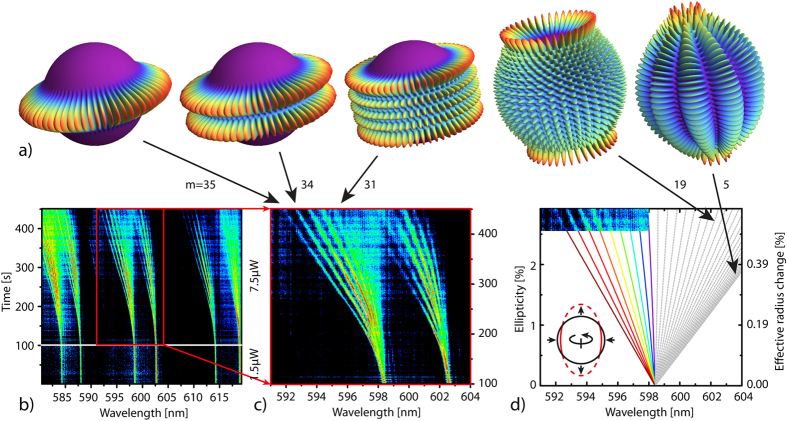
(**a**) Calculated electric field distribution on the sphere’s surface for the same angular momentum number 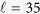 and for selected azimuthal mode numbers *m*. (**b**) Colour plot of the spectral time evolution of the experimentally observed resonances. Colouring from black to red represents low to high intensities. Under laser irradiation with 7.5 *μ*W power, the modes begin to split up. (**c**) Magnification of the region outlined in (**b**). (**d**) Calculated mode splitting using equation (1) and assuming a prolate distortion of the sphere, which increases linearly in time. Dashed lines represent modes with 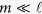, which are not visible in the experiment. The overlay in the upper left corner gives a direct comparison with the experimental data.

The spectrum in Fig. 1(c) was taken after 100 s illumination with 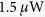, followed by 150 s optical excitation with 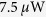. The complete time evolution of the mode splitting is shown in Fig. 2(b), using colour to represent intensity. For 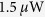 (below the white line) only a negligible change in the spectra is observed. At higher excitation power, the lines split up and their separation continuously increases. In Fig. 2(c), which shows an enlarged view of the 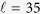 TE and 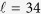 TM modes, it can also be seen that the splitting is the largest for the short wavelength lines.

Before the splitting occurs, the TE and TM modes in a sphere are 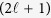-fold degenerate^11^, with respect to the azimuthal mode number 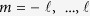. To visualize the significance of the azimuthal mode number *M*, Fig. 2(a) shows the absolute value of the electric field distribution at the sphere’s surface, calculated for the 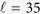 TE mode and different *m*’s, using the solutions to Maxwell’s equations within a dielectric sphere^8^. For 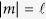, the electric field is mainly confined in the equatorial plane. For 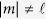, there are 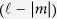 nodes along the polar angle, spreading the field across the sphere (see Fig. 2(a)). With decreasing 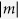, the field maxima shift towards the poles, which will become important for the interpretation of the experimental data below.

All these field distributions correspond to the same resonance frequency, but this degeneracy is lifted when the spherical symmetry is broken. This has been demonstrated, e.g., by deforming liquid droplets^12^,^13^ and by merging two spheres into one^14^. To account for our experimental observation, we assume that, in first approximation, the laser excitation induces an ellipsoidal distortion of the sphere, leading to a spheroid. According to perturbation theory calculations by Lai *et al.*^11^, the modes are then no longer degenerate and the *m*-dependent frequencies can be obtained from


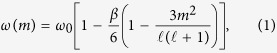


with the ellipticity 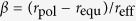, the polar and equatorial radii 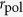 and 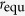, respectively, and the effective radius 
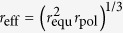
. Note that the frequency of the unperturbed sphere, 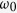, is dependent on 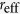, so that for a decreasing (increasing) change in volume, the whole multiplet shifts to shorter (longer) wavelengths. Thus, a continuous volume change will tilt the multiplet. With the radius 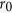 and the frequency 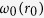 of the unperturbed sphere, determined as described above, it is now possible to model the splitting in order to assign the different lines in the multiplet to their corresponding polar mode number *m*.

We would like to point out that all considerations regarding the geometry are valid in terms of the *optical* path length. The optical path length in any dielectric body is governed by both the geometric distance and the index of refraction. Therefore, a local change of the dielectric function would be indistinguishable from an actual geometric shape change. In terms of the optical properties, both mechanisms would have the exact same effect, so that none of the considerations made here would be affected. For brevity, in the following, we use the term “shape change” to indicate the geometric change in terms of the *optical* path length.

It follows from equation (1) that the mode splitting is largest between 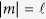 and 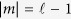 and is monotonically decreasing with decreasing 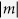. Since, experimentally, the largest splitting is observed on the short wavelength side of the multiplet, it can be concluded that the mode with the shortest wavelength corresponds to 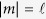. From this, in turn, it follows that the shape distortion induced by the optical irradiation is prolate (*β* > 0) rather than oblate (*β* < 0).

Taking from the experimental data the unperturbed frequency 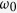 as well as the frequencies 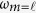 and 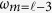 after 450 s of illumination, we obtain the polar and equatorial radii of the distorted sphere from a fit to equation (1). The modes 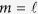 and 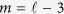 were chosen, because they correspond to the most separated frequencies that could still be precisely identified in the spectra. We find a polar distortion 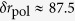 nm and an equatorial distortion 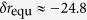 nm for the sphere investigated here (radius 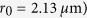, which corresponds to an ellipticity 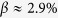. This justifies the use of equation 1, which is only valid for small excentricities. Furthermore, the change in volume and effective radius are 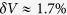 and 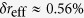, respectively. This allows us to calculate the entire mode evolution, assuming a linear change of both the equatorial and the polar radii in time, see Fig. 2(d). The results are in good agreement with the modes that can be discerned in the recorded spectra, in particular for those times when the experimental mode splitting is linear in time. This is also demonstrated by the overlay of the experimental and theoretical data in the top of Fig. 2(d). Note that the smallest splitting that can be well resolved in the experiment (around *t* = 200 s in Fig. 2(c)) corresponds to an equatorial distortion of 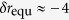 nm or, in terms of the wavelength, 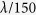.

A question that remains is why only modes with 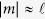 appear in the spectra. In order to explain this, we have to consider that in the experiments the spheres are supported by a substrate. We choose our coordinate system, so that the equatorial plane of the sphere is parallel to the supporting substrate and the contact point is at the “south pole”, i.e. at the polar angle 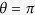. As the calculated electric field distribution in Fig. 2(a) shows, the mode with 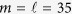 (leftmost panel) is not affected by the contact point between the sphere and the substrate, because the electric field becomes vanishingly small near the poles. This, however, changes with decreasing *m*. As exemplified by the calculated modes with 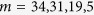, the electric field distribution spreads further and further towards the poles. This will lead to an increased leakage at the contact point into the substrate and, therefore, to an effective attenuation of the resonances with lower 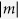.

**Figure 3:**
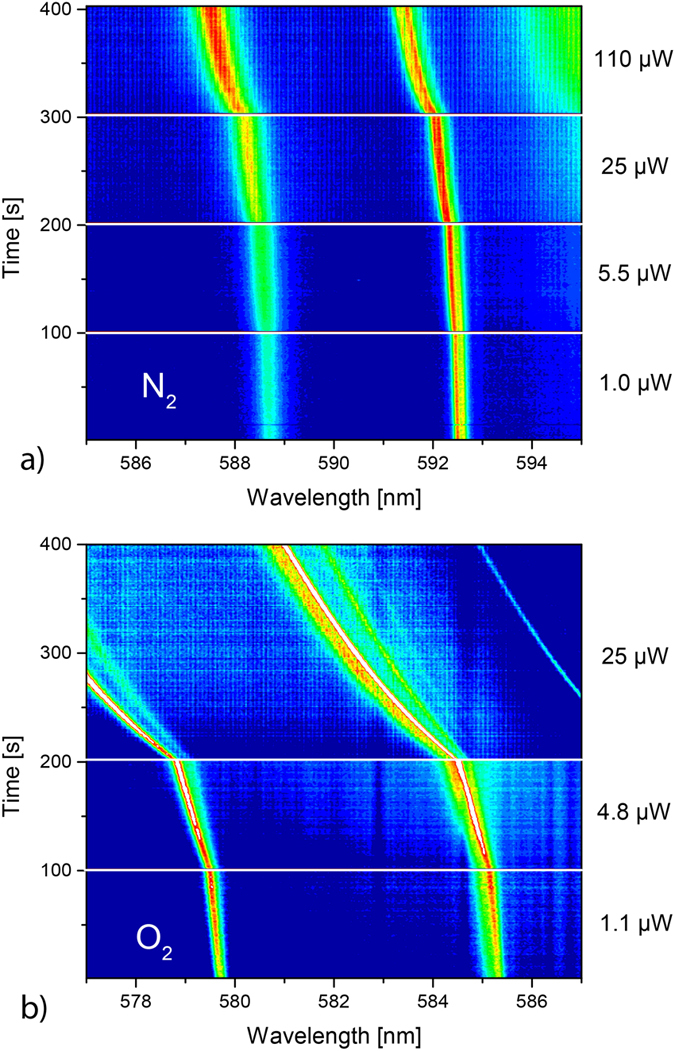
(**a**) Normalized spectral time evolution of whispering gallery modes in a sphere under nitrogen atmosphere. (**b**) Normalized spectral time evolution of whispering gallery modes in a sphere exposed to pure oxygen. Splitting into a multiplet is clearly visible, indicating that photooxidation is the origin for the degeneracy lifting.

In the following, we would like to discuss the mechanism that leads to the deformation of the polymer sphere. Obviously, the distortion is optically induced, and dependent on both the power and the duration of the illumination. To further elucidate the process that leads to the shape change, we have subjected the spheres to different environments during optical excitation. Figure 3(a) exemplarily shows a colour plot of a measurement under nitrogen atmosphere for laser powers exceeding those used in Fig. 2 by more than an order of magnitude. Within the experimental resolution, only a single mode is apparent and no splitting into a multiplet is observed, even for laser powers up to 1 mW (not shown here). Thus, no breaking of the spherical symmetry takes place. We attribute the (irreversible) small shift and the broadening that can be seen in Fig. 3(a) to residual gas contamination, e.g. oxygen (see below). Similar experiments under vacuum or water vapour-saturated nitrogen atmosphere (see supplementary information) did not reveal any mode splitting, either. On the other hand, oxygen in the environment during light exposure does lead to a degeneracy lifting, as Fig. 3(b) depicts. Thus, we attribute the splitting to a laser induced oxidation at the surface of the sphere. This conclusion is further supported by the fact that the observed splitting is irreversible, which indicates a chemical alteration of the material. It also excludes alternative explanations, such as the non-linear Kerr effect^15^. From the simulation it can be deduced that the sphere transforms into a prolate spheroid with a reduced equatorial radius and an increased polar radius. This anisotropic shape change is in agreement with the fact that we only observe modes with large *m*. These modes travel along the equator of the sphere (see Fig. 2(a)) so that the photooxidation effect will be most prominent in the equatorial plane. Photooxidation is known to change the refractive index of related polymers^16^,^17^,^18^. Furthermore, ellipsometric measurements on thin films, made from the same polymer as the spheres, clearly show a reduction in the refractive index after illumination under ambient conditions (see supplementary information). This will decrease the equatorial optical path length and can thus explain the observed ellipticity. However, an additional geometric shape change, induced, e.g., by a shrinkage of the polymer, may also contribute to the overall shape change.

Photooxidation of *π*-conjugated polymers is usually considered to be detrimental^19^,^20^. The present study, however, shows that it can also be a tool for *in-situ* modification of the shape and/or the index of refraction of optically active polymer structures. The fact that the whispering gallery mode linewidth does not increase as a result of photooxidation indicates that the excellent optical quality of the investigated polymer will not deteriorate during the photooxidation process.

## Summary

We have demonstrated that tailored *π*-conjugated copolymers are excellent starting materials for simple, yet effective fabrication of nearly perfect spherical optical resonators with Q-factors up to 10^4^. The high Q-factors achieved allow us to resolve minute changes in the symmetry properties of the resonators, which can be optically induced. We find a distinct splitting of both the TE and TM whispering gallery modes (which are highly degenerate in a perfectly spherical resonator) into a multiplet of lines with different azimuthal mode numbers *m*. From a comparison with a theoretical treatment of a weakly distorted sphere, we are able to deduce that, in terms of the optical path length, the resonator transforms into a prolate spheroid with a reduction of the equatorial radius 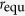. Changes in 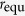 down to 4 nm can be resolved, corresponding to 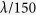. Investigations under various gaseous environments suggest that photooxidation of the polymer is the origin of the optically induced shape change.

Our findings show that self-assembled *π*-conjugated polymer spheres can have excellent optical properties, which are *in-situ* tunable. This makes them versatile building blocks for more sophisticated optically active structures, such as coupled resonators, photonic crystals, sensors, and detectors.

## Methods

The polymer, poly[(9,9-dioctylfluorene-2,7-diyl)-(5-octylthieno[3,4-c]pyrrole-4,6-dione-1,3-diyl)], was synthesized according to ref. 21. The number-average molecular weight and polydispersity index are 16200 g/mol and 2.10, respectively. The microspheres were produced using the vapour diffusion method^5^,^22^. The polymer was dissolved in chloroform (0.5 mg/mL) and methanol vapour was gradually diffused into the solution. The reduced solubility lead to slow polymer precipitation and the formation of spheres with a few micrometers in diameter (see Fig. 1, upper inset). The resulting dispersion was drop cast onto quartz glass substrates 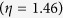 and dried under vacuum.

The sample was placed inside a vacuum chamber to measure time dependent PL-spectra under various gaseous atmospheres and different laser excitation powers. Single, well separated spheres were identified and individually characterized with a micro-PL setup, using a 532 nm 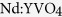 solid-state laser with a spot size of less than 1 *μ*m. The PL was collected by a 50× long-distance objective with a numerical aperture of 0.5 and detected with a liquid nitrogen cooled CCD camera, attached to a 0.5 m Czerny-Turner monochromator with a 1200/mm grating.

## Additional Information

**How to cite this article**: Braam, D. *et al.* Optically induced mode splitting in self-assembled, high quality-factor conjugated polymer microcavities. *Sci. Rep.* **6**, 19635; doi: 10.1038/srep19635 (2016).

## Supplementary Material

Supplementary Information
