# Supplementary material for: Optically induced mode splitting in self-assembled, high quality-factor conjugated polymer microcavities
Source: Sci Rep. 2016 Jan 19;6:19635. doi: 10.1038/srep19635 (PMC4725981; doi:10.1038/srep19635)
Supplement: Supplementary Information [file srep19635-s1.pdf]

Supplementary Information

Optically induced mode splitting in self-assembled, high quality-factor conjugated polymer microcavities

Daniel Braam*, Soh Kushida, Robert Niemöller, Günther M. Prinz, Hitoshi Saito, Takaki Kanbara, Junpei Kuwabara, Yohei Yamamoto, and Axel Lorke

*To whom correspondence should be addressed.
Email: Daniel.Braam@uni-due.de

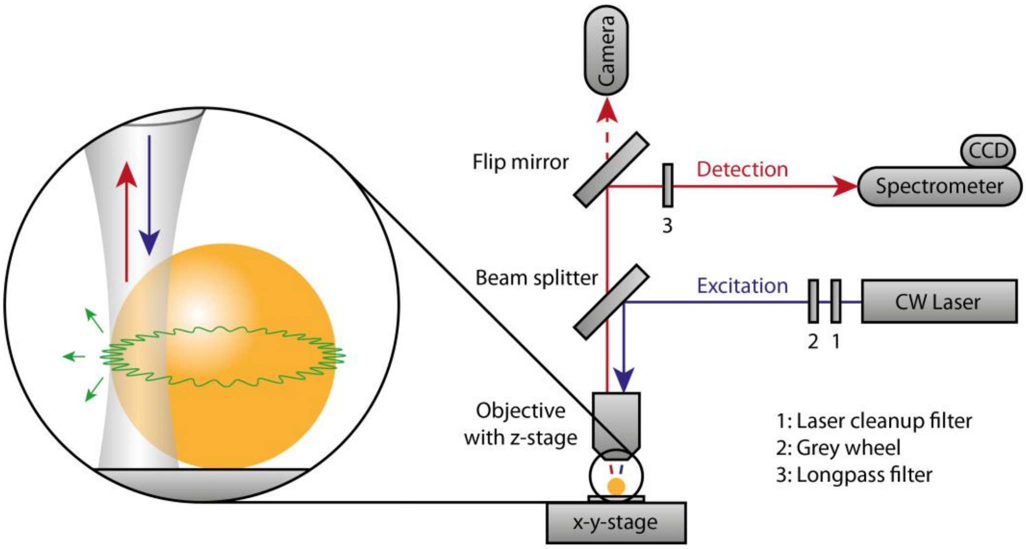

Supplementary figure S1: Schematic drawing of the PL setup with a magnification of the excitation method, indicating the light path around the equator.

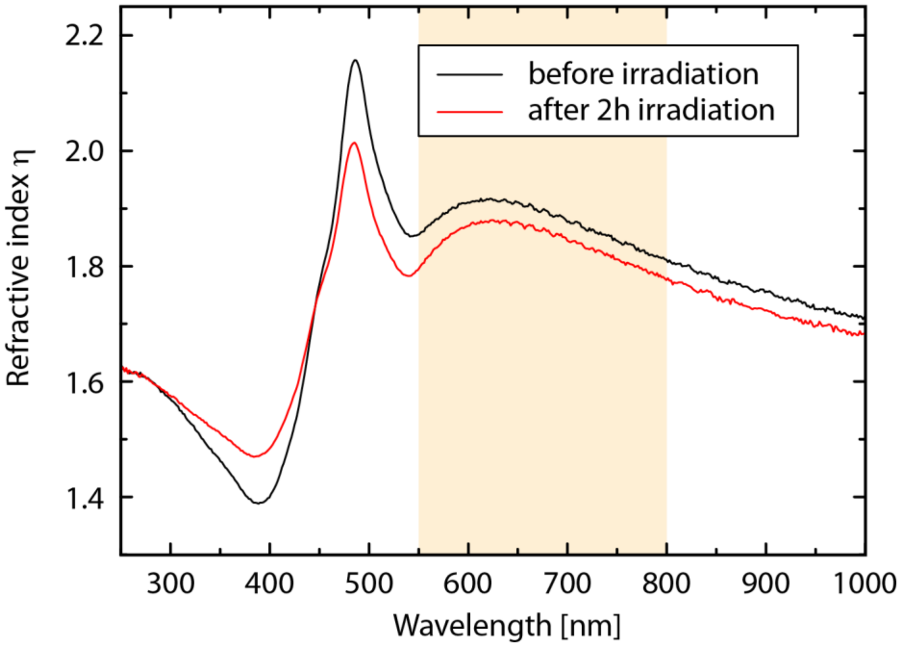

Supplementary figure S2: Refractive index η before and after 2h of irradiation under ambient conditions. Above 450 nm, a distinct decrease in η is observable.

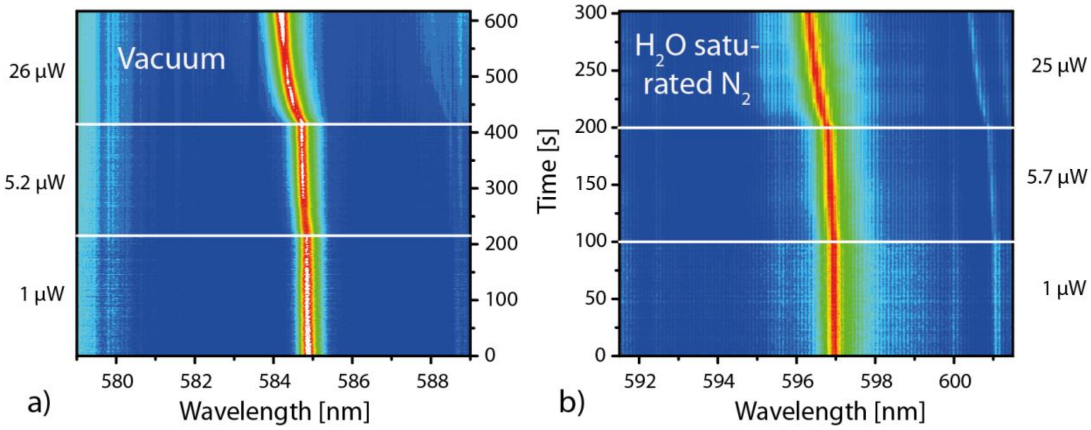

Supplementary figure S3: Normalized spectral time evolution of WGMs in single spheres under vacuum (a) and under H_2O saturated N_2 atmosphere (b), which show the same development as spheres illuminated under nitrogen atmosphere (see figure 3 of the main paper).
